# Monitoring the Outcome of Phonosurgery and Vocal Exercises with Established and New Diagnostic Tools

**DOI:** 10.1155/2020/4208189

**Published:** 2020-01-23

**Authors:** Matthias Seipelt, Andreas Möller, Tadeus Nawka, Ute Gonnermann, Felix Caffier, Philipp P. Caffier

**Affiliations:** ^1^Department of Audiology and Phoniatrics, Charité-University Medicine Berlin, Campus Charité Mitte, Chariteplatz 1, D-10117 Berlin, Germany; ^2^Max-Planck Institute for Plasma Physics, Wendelsteinstraße 1, D-17491 Greifswald, Germany; ^3^ENT Department, University of Greifswald, Fleischmannstraße 8, D-17475 Greifswald, Germany

## Abstract

Instrument-assisted measuring procedures expand the options within phoniatric diagnostics by quantifying the condition of the voice. The aim of this study was to examine objective treatment-associated changes of the recently developed vocal extent measure (VEM) and the established dysphonia severity index (DSI) in relation to subjective tools, i.e., self-evaluation via voice handicap index (VHI-12) and external evaluation via auditory-perceptual assessment of hoarseness (*H*). The findings for *H* (3 raters' group assessment), VHI-12, DSI, and VEM in 152 patients of both sexes (age range 16–75 years), taken before and 3 months after phonosurgery or vocal exercises, were compared and correlated. Posttherapeutically, all of the recorded parameters improved (*p* < 0.001). The degree of *H* reduced on average by 0.5, the VHI-12 score sank by 5 points, while DSI and VEM rose by 1.5 and 19, respectively. The correlations of these changes were significant but showed gradual differences between *H* and VHI-12 (*r* = 0.3), *H* and DSI (*r* = −0.3), and *H* and VEM (*r* = −0.4). We conclude that all investigated parameters are adequate to verify therapeutic outcomes but represent different dimensions of the voice. However, changes in the degree of *H* as gold standard were best recognized with the new VEM.

## 1. Introduction

The human voice is a very complex phenomenon that is difficult to quantify [1–3]. According to the basic protocol of the European Laryngological Society, a comprehensive assessment of vocal function can be gained using a multidimensional diagnostic approach [4]. Several measurements are recommended for voice evaluation, comprising subjective procedures such as self-assessment of the voice and external auditory-perceptual judgment, as well as objective procedures such as voice range profile (VRP) measurements, acoustic-aerodynamic analysis, and videolaryngostroboscopy (VLS).

In order to quantify the self-experienced extent of a vocal problem, the subjective impairment can be assessed using standardized questionnaires [5, 6]. The original voice handicap index (VHI) consists of 30 questions addressing functional, physical, or emotional aspects in the context of dysphonia [7]. Shorter VHI versions were designed because many patients perceive the answering of 30 questions tedious and partly redundant [8]. The 9-item VHI-9i and the 12-item VHI-12 had been created after original item reduction based on factor analysis and test-retest validation. They represent reliable, commonly used questionnaires with improved acceptance and practicability in clinical routine [9]. Regardless of the subjective self-evaluation, the examiner's auditory perception of the patient's voice is considered in many medical studies as the gold standard for voice assessment [10–12]. Different evaluation systems were developed, assessing various parameters including the perceived roughness (*R*), breathiness (*B*), and the overall grade of hoarseness (*H*). The application of the RBH scale is considered to be reliable, particularly when group assessments are used for further analysis [13–15].

**Figure 1:**
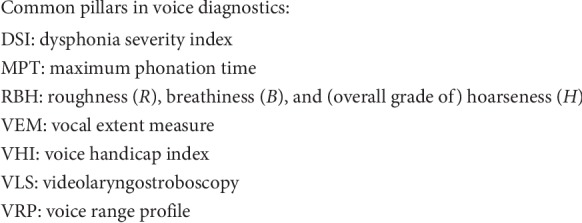
List of common abbreviations in voice diagnostics.

The inclusion of instrument-assisted measurement procedures can support and usefully expand the diagnostic investigation by objectively quantifying the current condition of the voice [16, 17]. The established dysphonia severity index (DSI) is calculated as a weighted combination of the highest possible fundamental frequency (*F*0_max), the lowest phonation intensity (*I*_min), maximum phonation time (MPT), and jitter [18]. Since the DSI quantifies dysphonia as a negative criterion and involves the risk of inaccurate results due to its multidimensional acquisition, we recently developed the one-dimensional vocal extent measure (VEM) for objective VRP evaluation [19]. The VEM quantifies the subject's dynamic performance and frequency range and is calculated as a relation of area and perimeter of the VRP. The VEM describes the vocal abilities and enables a classification of voice performance as a positive criterion [20]. A list of common abbreviations in voice diagnostics is given in Figure 1.

For comprehensive documentation of vocal status and treatment, it is necessary to ensure that changes in voice quality can be adequately identified. For this purpose, the measurement data used must be sensitive to the slightest changes in voice quality, and the registration equipment must be able to detect them. In order to investigate the suitability of objective and subjective parameters for the assessment of vocal improvement after phonosurgery and vocal exercises, the changes in DSI and VEM values should be monitored and compared with those of the subjective auditory perception via RBH and VHI-12.

## 2. Materials and Methods

A total of 152 patients with various voice problems underwent therapy in a clinical prospective study. The trial was conducted in accordance with the Declaration of Helsinki and on approval by the local ethical review board. All data were collected at the pretherapeutic visit and three months after the intervention. According to the diagnosed clinical pathology and the previous course of the disease, the patients received either surgical treatment or conservative vocal exercises. Logopedic voice therapy was conducted by qualified speech therapists and included 20 sessions (2 times per week, for 45 minutes). Phonomicrosurgery was performed by 3 experienced senior phonosurgeons via direct microlaryngoscopy in general anesthesia (TIVA with propofol/remifentanil).

Various established examination instruments were applied to evaluate the treatment outcome. Digital VLS was performed using a high-resolution rigid video laryngoscope (10 mm; 70°) with an integrated microphone (XION medical, Berlin, Germany) [21]. Laryngoscopy served to discriminate between organic dysphonia and functional dysphonia. Stroboscopy visualized the vocal fold vibrations during phonation and indicated impairment by showing reduced/absent mucosal wave propagation or reduced/eliminated phonatory vibration.

The LingWAVES program (WEVOSYS, Forchheim, Germany) was used for standardized registration of the VRP and acoustic-aerodynamic analysis. Several acoustic and aerodynamic parameters were recorded, such as I_min, F0_max, MPT, and jitter. Based on the defined combination of these parameters, the DSI was calculated to classify the voice into nondysphonic (≥4.2) versus mildly (<4.2 to ≥1.8), moderately (<1.8 to ≥−1.2), or severely (<−1.2) dysphonic [22]. In addition, the VEM as a recently introduced new diagnostic tool for the objective assessment of vocal capacity was computed [19, 23]. VEM calculation was done after VRP measurement by a proprietary software program (AVA) which can extract various other parameters from the VRP, thereby enabling VRP comparisons [24]. The VEM multiplies the VRP area by the quotient of the theoretical perimeter of a circle with the VRP surface area and the actual VRP circumference. The mathematical derivation of the equation of this measure is explained elsewhere [19]. The VEM quantifies the dynamic performance and frequency range of the voice by a one-dimensional, interval-scaled value without unit, typically between 0 and 120. These limits may be exceeded at both ends (VEM_min_ ≥ −150; VEM_max_ ≤ 150), describing a small vocal capacity by a small VEM and a large VRP by a high VEM.

The VHI-12 was applied for the patient's subjective self-assessment of the own voice [9]. Study participants rated all 12 questions on a scale from 0 to 4 (0: never, 1: almost never, 2: sometimes, 3: almost always, 4: always), followed by one question concerning the overall voice impairment at the present time (VHIs) on a scale from 0 to 3 (0: normal, 1: mild, 2: moderate, 3: severe). The VHI-12 total score allowed an impairment-related severity classification (0–7: no dysphonia, 8–14: mild dysphonia, 15–22: moderate dysphonia, 23–48: severe dysphonia).

External auditory-perceptual voice evaluation was assessed using the RBH system when the patients were reading the standardized text “The north wind and the sun” (German version). The perceived roughness (*R*), breathiness (*B*), and overall grade of hoarseness (*H*) of the patients voices were scored on a scale from 0 to 3 (0: not existing, 1: mild, 2: moderate, 3: severe) by three experienced examiners (one phoniatric physician, one clinical linguist, and one biomedical engineer). To enhance the evaluation objectivity, all audio recordings were rated independently in one session after being shuffled and blinded regarding the patient assignment and pre-/posttherapeutic status. The degree of *H* served as a gold standard to provide an indication of the therapy success.

The outcome analysis was based on pre- and posttherapeutic voice function diagnostics and VLS. The parameters *H*, VHI-12, VHIs, DSI, and VEM were compared with each other before and three months after the intervention, as well as their changes. It was tested whether the therapy resulted in a significant difference in the parameters measured. In addition, the measurement data were correlated with each other before and after therapy, as were the respective changes. Statistical methods applied were the calculation of Spearman's rank-order correlation coefficients (*r*), as well as the paired *t*-test. The level of significance was set at *α* = 0.05.

## 3. Results

Altogether, 152 patients were examined before and after therapeutic treatment: 102 females (17–70 years, median 48) and 50 males (16–75 years, median 42). A total of 304 data sets were collected. Subjects of both sexes were comparable in terms of age, sociodemographic characteristics, hoarseness level (*H*), and underlying pathologies. Sixty-six individuals (43%) used their voice in a nonprofessional manner (e.g., clerks, IT-specialists, and laborers), whereas 86 patients (57%) had a high vocal strain in their profession (e.g., teachers, actors, and singers). Pretherapeutically, the patients exhibited various clinical disorders. VLS revealed in 101 subjects (66%) organic diseases at vocal fold level. Classification of the resulting organic dysphonia according to the underlying pathology showed in 41 patients (27%) diseases of the lamina propria (e.g., nodules, polyps, cysts, and edema), in 24 patients (16%) movement disorders (vocal fold paralysis, spasmodic dysphonia), in 19 patients (12%) diseases of the epithelium (e.g., leukoplakia, hyperkeratosis, carcinoma, and papillomatosis), and in 5 patients (3%) arytenoid pathologies (granuloma). Fifty-one participants (34%) had normal laryngeal anatomy but suffered from a vocal load-induced functional dysphonia. Altogether, 46 subjects (30%) had initially no hoarseness (H0), including 29 patients with functional dysphonia, 13 patients with small glottal findings (marginal edema, nodules, and leukoplakia), and 4 patients with pathologies distant from the vocal fold level (arytenoid granuloma). A summary of relevant pretherapeutic patient characteristics, pathology classification according to Rosen and Murry [25], and a listing of all diagnoses are shown in Table 1.

**Figure 2:**
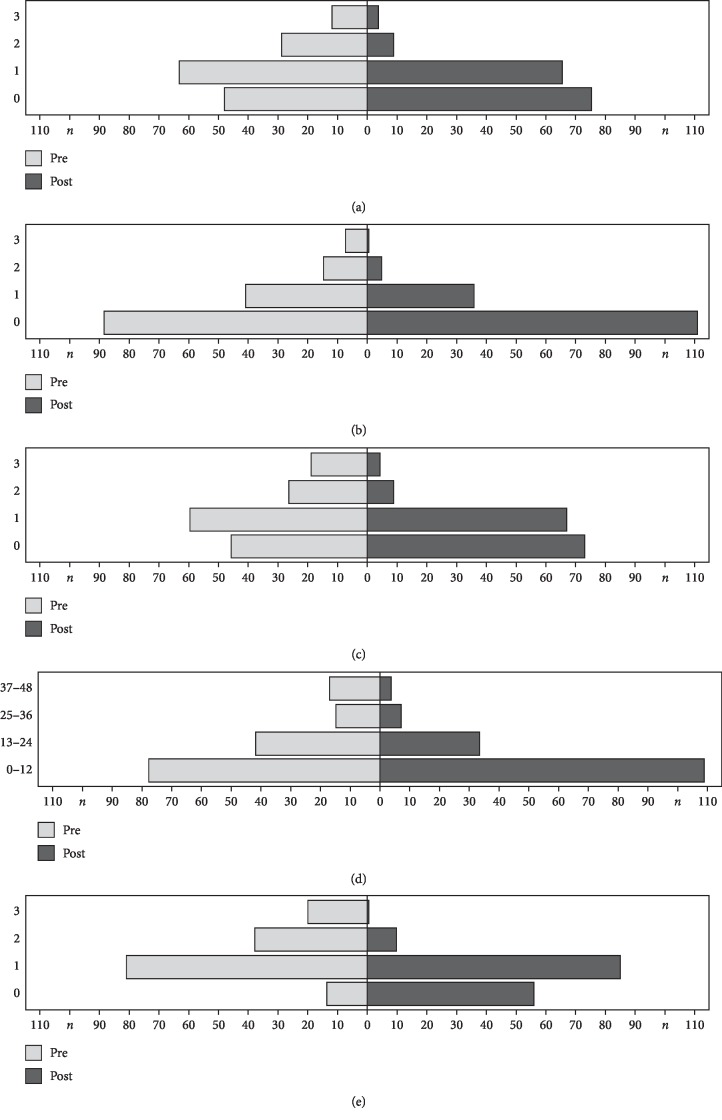
Subjective vocal parameters: (a) *R*, (b) *B*, (c) *H*, (d) VHI-12, and (e) VHIs before treatment (light grey, left columns) and after treatment (dark grey, right columns). The abscissae show the number of patients (*n*), and the ordinates represent the scales of the RBH system and VHIs system (0–3) as well as the VHI-12 score.

Posttherapeutically, all investigated vocal parameters had improved. Regarding subjective evaluation, the mean RBH status exposed less roughness, breathiness, and overall grade of hoarseness (*p* < 0.001). *H* decreased from 1.2 to 0.7, changing in most patients from H1 to H0 (*n* = 29; i.e., 19%) and from H2 to H1 (*n* = 22; i.e., 14%). The VHI-12 reduced on average from 15 to 10, corresponding to a self-assessed improvement from moderately to mildly impaired (*p* < 0.001). Respectively, the overall VHIs score sank from 1.4 to 0.7, changing most often from mild voice impairment to normal (*n* = 44; i.e., 29%) and from moderate to mild voice impairment (*n* = 35; i.e., 23%).  Figure 2 summarizes the mean pre- and posttherapeutic data in all patients for the investigated subjective vocal parameters using column diagrams.

**Figure 3:**
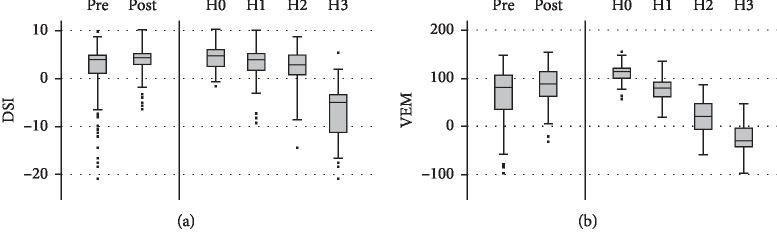
Objective vocal parameters: (a) DSI and (b) VEM before and after treatment, as well as their distribution according to the degree of *H*. The boxplots display the median, quartiles, range of values covered by the data, and any outliers (single spots).

A comparison of objective parameters revealed for the DSI a mean increase from 2.2 to 3.7, showing significant improvement (*p* < 0.001) that remained at the level of mild dysphonia. The VEM rose from 60 to 79, reflecting voice improvement with significantly enhanced vocal capacity (*p* < 0.001).  Figure 3 illustrates the pre- and posttherapeutic data for both objective voice parameters using boxplots. It indicates additionally the different distribution of DSI and VEM in relation to the degree of *H*.

**Figure 4:**
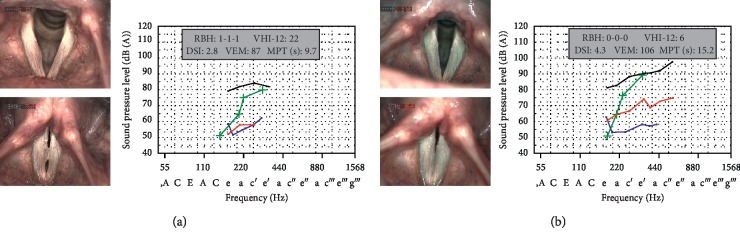
Example of phonosurgery-induced changes of laryngeal and vocal findings in a 49-year-old female dental assistant suffering from persisting dysphonia and dysodia. (a) Preoperative VLS shows a marginal edema of the right vocal fold with a glottal gap during phonation, asynchronous oscillations, and impaired mucosal wave propagation. The preoperative VRP pattern displays envelope curves for the loudest (black lines) and softest (blue lines) singing voice and for the speaking voice at different vocal intensity levels (green lines) with little dynamic and frequency ranges. The singer's formant levels (red lines) are low, characterizing the impaired concentration of acoustic energy by resonator amplification of certain frequency ranges in the vocal tract. The values of all objective voice parameters (DSI: dysphonia severity index; VEM: vocal extent measure; MPT: maximum phonation time) and subjective voice parameters (RBH: roughness, breathiness, overall grade of hoarseness; VHI-12: twelve-item voice handicap index) are reduced. (b) Three months after phonomicrosurgical removal of the edema, the treated vocal fold shows a straight margin. The glottal closure is complete, and the oscillations have normalized (mucosal wave propagation regular and symmetric). The patient reveals higher dynamic and frequency ranges of speaking and singing voice with considerably improved objective and subjective parameters.

A comparison of treatment groups revealed that phonosurgery had the largest impact on voice function with higher numerical improvement of subjective and objective parameters. A patient example demonstrating phonosurgery-induced changes of laryngeal and vocal findings is presented in Figure 4.

To evaluate the extent of treatment-related benefits, Table 2 shows the mean differences between pre- and posttherapeutic values and the 95% confidence intervals for them. The numeric outcome of the values after conservative logopedic therapy was much smaller, but the vocal capabilities improved in most patients, too. Furthermore, Table 2 displays the pre- and posttherapeutic comparison concerning both dysphonia groups (functional/organic) and all pathology classification subgroups. In general, age and gender had no significant influence on the treatment outcome.

The correlation of the parameters *H*, VHI-12, VHIs, DSI, and VEM with each other showed a significant (*p* < 0.01) but weak to moderate linear relationship. The strength of the relationship changed only slightly due to the therapy. However, the DSI proved an exception in this regard. While the DSI data before treatment showed a moderate negative relationship with *H* (*r* = −0.4) and moderate positive relationship with VEM (*r* = 0.6), these correlations decreased considerably after therapy, revealing weaker relationships for *H* (*r* = −0.3) and VEM (*r* = 0.3). The weak relationship between DSI and VHI-12 as well as DSI and VHIs did not show relevant changes posttherapeutically. In contrast, the VEM correlated with the VHI-12 at *r* = −0.4 and with *H* at *r* = −0.7, revealing moderate and strong negative relationships, irrespective of the therapy status. Furthermore, *H* and VHI-12 correlated after therapy at *r* = 0.4, and *H* and VHIs at *r* = 0.5.

The investigation of therapy-induced changes (Δ) in the individual measurement data indicated that correlations of these changes resulted in rather small coefficients for all parameters. The relationship between ΔDSI and Δ*H* was *r* = −0.3 (*p* < 0.01). The ΔDSI showed no significant relationship to ΔVHI-12 (*r* = −0.04) and ΔVHIs (*r* = −0.09). The relationship between ΔVEM and Δ*H* was *r* = −0.4 (*p* < 0.001). In contrast to DSI, ΔVEM revealed also a significant relationship to ΔVHI-12 and ΔVHIs (*r* = −0.2 each, *p* < 0.01). Besides, Δ*H* and ΔVHI-12 correlated at *r* = 0.3, and Δ*H* and ΔVHIs at *r* = 0.4 (*p* < 0.01). Finally, the relationship between ΔDSI and ΔVEM was moderate at *r* = 0.5 (*p* < 0.01). A summary of all correlation results can be found in Table 3.

## 4. Discussion

It was possible to show that all parameters under investigation reacted to the therapy and improved on average, thus presenting their general suitability for documentation of the therapeutic process. However, due to the individual construction and intention, each objective and subjective parameter performed differently. The established DSI represents a weighted sum of I_min, F0_max, MPT, and jitter [18] and therefore integrates parameters of VRP, aerodynamic, and acoustic measures. As assumed, most of our patients showed DSI values ranging from −5 to 5, whereby −5 corresponded to a very dysphonic voice and 5 to a perceptual normal voice. Due to the special structure of our patient cohort including a considerable number of elite vocal performers and subjects with extremely dysphonic voices, more study participants than expected (34%) had initial DSI values which exceeded these boundaries at both ends. After therapy, the mean DSI measurement data did not improve by an average of one degree of severity [22] but still showed a significant increase. This confirms previous studies, which describe the DSI as a useful parameter to measure the severity of dysphonia and the improvement after therapy [26–29]. However, various studies could show that the DSI is influenced by differences in measurements of the registration programs as well as by age or gender [19, 30–32]. Therefore, we developed and investigated the VEM as a new objective vocal parameter unimpaired by these interacting factors.

The VEM calculation is based on the size and shape of the VRP, instead of gathering data from a combination of different objective parameters [19, 24]. Most of our patients showed VEM values between 0 and 120. As expected, these limits were exceeded: (1) at the upper end in functionally impaired singers with professionally trained “great” voices and very large VRP, and (2) at the lower end in extremely dysphonic and nearly aphonic voices with very small VRP. These findings support the underlying idea, during the construction of this vocal parameter, that the ideal VRP should not show abrupt differences in the dynamic range of notes produced by the patients along with their frequency range [24]. A well-balanced dynamic extent approximated the optimal VRP shape to a circle where the area is the biggest for a given perimeter compared to other geometric figures [19]. Our results confirmed that larger and “smoother” VRP without relevant “jumps” in intensity achieved higher values. Vocal capacity quantified in this way, i.e., as a relation of area and perimeter of the VRP, showed a very distinct increase during the course of therapy in our study participants. This is in line with the results of the very few phonosurgical studies assessing VEM values in patients with Reinke's edema [20], vocal fold polyps [23], and nodules [33]. All of these investigations observed significantly increased VEM values after treatment.

Concerning subjective parameters, the VHI-12 successfully quantified the self-experienced extent of the vocal problem in our patients. Due to therapy, most of them rated an improvement from moderately to mildly impaired. These results correspond to other studies using VHI questionnaires for the investigation of surgical and conservative treatment success in organic dysphonia and functional dysphonia [20, 23, 33–36]. Our overall impression supports the general acknowledgment that short-form VHI versions represent reliable instruments with excellent acceptance and practicability in clinical routine [8, 9]. Compared to previous investigations, the examiners' auditory perception was the main indicator in our study for the assessment of therapy success [10–13]. We consider the RBH system to be reliable, particularly in case of evaluation by group assessments [14, 15, 23]. The mean RBH status of our patients' voices revealed significantly less roughness, breathiness, and overall grade of hoarseness. These results also confirmed the outcomes of former studies [23, 33–35]. Additionally, our analysis of the degree of *H* in relation to the objective parameters DSI and VEM revealed a better representation and graphical distinction of the auditory-perceptual assessment via the VEM. This is a new and important study finding which was confirmed in our investigations of correlation.

Correlations between the changes in individual parameters are able to show how well the improvement in one measurement value is reproduced by another measuring procedure. The generally weak correlations in our results can be explained by the different approaches to the individual parameters and are likewise a manifestation of the additional information content of the respective measurement methods. A relatively high correlation between parameters confirms the success of the therapy from the different aspects of these parameters. Regarding DSI, only a weak negative correlation with *H* and no significant relationship to VHI-12 could be found. Overall, this implies that although the DSI seems suitable for indicating the success of therapy, the increase of DSI has very little to do with the improvement in the degree of hoarseness and with the patient's perception of the vocal problem. This is also seen in the decreasing correlation of the values for DSI and *H* after therapy. The changes in the VHI-12, on the other hand, had a weak relationship to the changes of the VEM. Moreover, changes in the VEM demonstrated a moderately negative relationship to the changes of *H*, which means that the VEM increases as hoarseness decreases. Thus, in addition to the quantification of vocal capacity, the VEM is validated by the auditory assessment. The novel numeric description of the VRP by means of the interval-scaled VEM provides the researcher with a diagnostic parameter which is suitable for monitoring the course of treatment.

While interpreting these results, some limitations of our study should be considered. First, the number of patients was too small and the cohort was too heterogeneous to examine comparably sized groups of *H* levels, pathology classification, or diagnosis-related subgroups. Therefore, there could be participation bias. Second, individual treatment recommendations depended on phoniatric indication and were based on comprehensive counseling related to clinical signs, symptoms, individual vocal requirements, abilities, and medical history. Nevertheless, at the end, the patients decided about the kind of intervention; thus, there may be selection bias. Third, our posttherapeutic follow-up of three months was too short to allow statements about the long-term outcome. Fourth, the investigated treatment modalities are often used in a combined mode to accelerate and optimize vocal improvement. We were not able to control whether patients after phonosurgery received hidden other therapies. Additional logopedic treatment or singing lessons are easily accessible and could influence the results especially in the recovery of operated patients. Therefore, there may be also performance bias. Finally, some well-known factors influencing the VRP registration have to be taken into account, such as the routine of the examiner, musicality and motivation of the patients, and the absence of generally accepted specifications regarding the number of registered tones. However, all VRPs were recorded by one experienced examiner under practically equal conditions, so that most of the mentioned factors can be ignored in this study.

Overall, our specific therapeutic outcomes confirmed the results of other studies investigating treatment effects in patients with various voice problems [33–39]. As expected, phonosurgery had the largest numeric impact on the improvement of vocal function. Conservative therapy provided smaller quantitative enhancements but often also qualitative vocal restoration with recovered artistic capabilities, particularly in singers with functional dysphonia. Logopedic training goals typically included reducing extrinsic laryngeal tension, using a relaxed laryngeal posture, and effective abdominal-diaphragmatic support for all phonation events [40]. Specific attention was given to the balance of respiratory forces, laryngeal coordination, and optimal filtering of the source signal via resonance and articulatory awareness [41, 42]. With this approach, also some of our patients with organic findings gained substantial voice improvement. As known from the literature, mainly younger patients with short duration of dysphonia and small benign pathologies of the lamina propria (e.g., vocal fold polyps, marginal edema) due to overuse benefitted from voice therapy [43–45].

## 5. Conclusions

The investigated parameters DSI, VEM, VHI, and RBH are all suitable for monitoring the course of voice treatment and adequate to quantify the outcomes of phonosurgery and logopedic vocal exercises. Correlation analysis confirms the clinical impression that DSI, VEM, VHI, and RBH represent different dimensions of the voice and are complementing objective or subjective measurements either for the evaluation of voice quality, vocal performance, or perceived vocal handicap. The VEM proves to be a comprehensible and easy-to-use parameter for objective VRP evaluation. Changes in the degree of hoarseness as gold standard were best recognized with the new VEM. Thus, in addition to the quantification of vocal capacity, the VEM is supported and validated by the auditory findings and provides an interval-scaled parameter for documentation.

## Figures and Tables

**Table 1:** Pretherapeutic patient characteristics.

Characteristics	No. of all patients	% of total group (*n* = 152)	No. of male patients	% of male group (*n* = 50)	No. of female patients	% of female group (*n* = 102)
*Gender*						
Male	50	33	—	—	—	—
Female	102	67	—	—	—	—

*Age*						
Years (mean ± SD)	45 ± 16	—	43 ± 17	—	45 ± 15	

*Main voice use*						
Nonprofessional	66	43	22	44	44	43
Professional	86	57	28	56	58	57
*Sociodemographic*						
Scholar	10	7	4	8	6	6
Student/apprentice	14	9	7	14	7	7
Employed	97	64	29	58	68	66
Unemployed	8	5	2	4	6	6
Pensioner	23	15	8	16	15	15

*Overall hoarseness level (H)*						
*H*0 (not existing)	46	30	12	24	34	33
*H*1 (mild)	6	40	21	42	39	38
*H*2 (moderate)	27	18	10	20	17	17
*H*3 (severe)	19	12	7	14	12	12

*Pathology classification* ^*∗*^						
Functional dysphonia	51	34	15	30	36	35
Organic dysphonia	101	66	35	70	66	65
Rosen I (epithelium)	19	12	10	20	9	9
Rosen II (lamina propria)	41	27	7	14	34	33
Rosen III (arytenoid)	5	3	3	6	2	2
Rosen IV (other)	36	24	15	30	21	21

*Organic diagnosis*						
Vocal fold paralysis	18	11.8	9	18.0	9	8.8
Vocal fold nodules	13	8.6	0	—	13	12.7
Vocal fold polyp	9	5.9	4	8.0	5	4.9
Reinke's edema	9	5.9	0	—	9	8.8
Laryngeal papillomatosis	8	5.3	3	6.0	5	4.9
Marginal edema	6	3.9	2	4.0	4	3.9
Spasmodic dysphonia	6	3.9	0	—	6	5.9
Contact granuloma	5	3.2	3	6.0	2	2.0
Vocal fold atrophy	5	3.2	4	8.0	1	1.0
Hyperkeratosis	4	2.6	3	6.0	1	1.0
Leukoplakia	4	2.6	3	6.0	1	1.0
Sulcus vocalis	3	2.0	2	4.0	1	1.0
Glottal carcinoma (pT1a)	3	2.0	1	2.0	2	2.0
Vocal fold cyst	3	2.0	1	2.0	2	2.0
Varix cordis	1	0.7	0	—	1	1.0
Laryngotracheal stenosis	1	0.7	0	—	1	1.0
Glottal web	1	0.7	0	—	1	1.0
Traumatic laryngeal fracture	1	0.7	0	—	1	1.0
Bamboo nodes	1	0.7	0	—	1	1.0

Unless otherwise specified, data expressed as number of patients and percentage of group. ^*∗*^Pathology classification according to Rosen and Murry [25], i.e., I: epithelium, e.g., leukoplakia, hyperkeratosis, CIS (=carcinoma in situ), carcinoma, and papillomatosis. II: lamina propria, e.g., Reinke's edema, polyps, cysts, scars, and vascular malformation. III: arytenoid, e.g., granuloma and infection. IV: other, including movement disorders, hypo-/atrophy, and malformation as e.g., sulcus or glottal web.

**Table 2:** Changes in vocal measures after treatment for all patients and separated for both intervention groups (logopedics/phonosurgery), both dysphonia groups (functional/organic), and all pathology classification subgroups according to Rosen and Murry [25].

	*H*	VHI-12	VHIs	DSI	VEM
Total group of patients (*n* = 152)	−0.5 (−0.6; −0.4)	−4.8 (−6.2; −3.3)	−0.7 (−0.8; −0.6)	1.5 (1.0; 2.0)	18.7 (13.4; 24.1)
Logopedic treatment group (*n* = 79)	−0.3 (−0.4; −0.2)	−1.4 (−2.6; −0.1)	−0.5 (−0.7; −0.4)	0.2 (−0.1; 0.5)	6.5 (1.6; 11.4)
Phonosurgery group (*n* = 73)	−0.8 (−1.0; −0.6)	−8.5 (−10.9; −6.0)	−0.9 (−1.1; −0.7)	2.8 (1.9; 3.7)	32.0 (22.9; 41.0)
Functional dysphonia group (*n* = 51)	−0.3 (−0.4; −0.2)	−0.3 (−1.4; 0.7)	−0.5 (−0.7; −0.4)	0.1 (−0.2; 0.4)	3.6 (0.1; 7.1)
Organic dysphonia group (*n* = 101)	−0.7 (−0.8; −0.5)	−7.0 (−9.0; −5.0)	−0.8 (−1.0; −0.7)	2.2 (1.5; 2.9)	26.4 (18.9; 33.9)
Rosen I subgroup (epithelium) (*n* = 19)	−0.7 (−1.2; −0.3)	−5.1 (−9.6; −0.6)	−1.0 (−1.3; −0.7)	2.0 (0.5; 3.6)	30.2 (10.1; 50.3)
Rosen II subgroup (lamina propria) (*n* = 41)	−0.6 (−0.7; −0.4)	−4.6 (−6.8; −2.4)	−0.7 (−0.9; −0.5)	1.2 (0.2; 2.1)	15.7 (7.3; 24.1)
Rosen III subgroup (arytenoid) (*n* = 5)	−0.1 (−0.4; 0.3)	−1.6 (−6.0; 2.8)	−0.3 (−0.9; 0.3)	0.2 (−0.8; 0.9)	2.5 (−11.8; 16.7)
Rosen IV subgroup (other) (*n* = 36)	−0.8 (−1.1; −0.5)	−11.5 (−15.7; −7.4)	−1.0 (−1.2; −0.7)	3.9 (2.6; 5.3)	39.9 (24.7; 55.1)

Data expressed as mean differences of preoperative and postoperative values (upper line), with 95% confidence intervals (lower line, in brackets). VEM = vocal extent measure; DSI = dysphonia severity index; VHI-12 = voice handicap index; VHIs = self-perceived impairment of voice at the present time.

**Table 3:** Results of correlation analysis.

		DSI	VEM	*H*	VHI-12	VHIs
Pretherapeutic	DSI	1	0.6	−0.4	−0.2	−0.3
	VEM	0.6	1	−0.7	−0.4	−0.3
	*H*	−0.4	−0.7	1	0.4	0.4
	VHI-12	−0.2	−0.4	0.4	1	0.6
	VHIs	−0.3	−0.3	0.4	0.6	1

	DSI	1	0.3	−0.3	−0.3	−0.3
	VEM	0.3	1	−0.7	−0.3	−0.4
Posttherapeutic	*H*	−0.3	−0.7	1	0.4	0.5
	VHI-12	−0.3	−0.3	0.4	1	0.7
	VHIs	−0.3	−0.4	0.5	0.7	1

	DSI	1	0.5	−0.3	−0.04 (ns)	−0.09 (ns)
	VEM	0.5	1	−0.4	−0.2	−0.2
Therapy-induced changes (Δ)	*H*	−0.3	−0.4	1	0.3	0.4
	VHI-12	−0.04 (ns)	−0.2	0.3	1	0.6
	VHIs	−0.09 (ns)	−0.2	0.4	0.6	1

All correlation coefficients were significant (*p* < 0.01), unless otherwise specified (ns = not significant).

## Data Availability

The datasets generated and analyzed for this study are not publicly available, as they were obtained from a proprietary database via a licensing agreement.
